# Effects of Massa Medicata Fermentata on the intestinal pathogenic flagella bacteria and visceral hypersensitivity in rats with irritable bowel syndrome

**DOI:** 10.3389/fphys.2022.1039804

**Published:** 2022-11-24

**Authors:** Zhaomeng Zhuang, Chen Huang, Yiguang Zhang, Bin Lv

**Affiliations:** ^1^ Zhejiang Chinese Medical University Affiliated Wenzhou Hospital of Integrated Traditional Chinese and Western Medicine, Wenzhou, China; ^2^ Department of Gastroenterology, The First Affiliated Hospital of Zhejiang Chinese Medical University (Zhejiang Provincial Hospital of Traditional Chinese Medicine), Hangzhou, China

**Keywords:** IBS-D diarrhoea-predominant IBS, TLR5, MMF, visceral hypersensitivity, intesetinal flora

## Abstract

**Objective:** To investigate the effect of Massa Medicata Fermentata (MMF) on the changes of pathogenic flagellar bacteria and visceral hypersensitivity in rats with diarrhea irritable bowel syndrome (IBS-D).

**Methods:** Thirty adult SD rats were randomly divided into normal control group (*n* = 10), model control group (*n* = 10), and MMF group (*n* = 10). Acetic acid enema combined with restraint stress was used to build the IBS-D visceral hypersensitivity model; Abdominal withdrawal reflex (AWR) test was used to assess the visceral sensitivity of rats; 16SrRNA sequencing was used to analyze the changes of intestinal bacteria in each group, and the content of pathogenic flagellated bacteria were quantitatively counted; The content of flagellin in colonic mucosa was detected by ELISA; TLR5 protein in colonic mucosa of rats was detected by Western Blot.

**Results:** After IBS-D modeling, the visceral sensitivity of rats was significantly higher in the model control group than that in the normal control group (*p* = 0.0061), while it was significantly decreased in MMF group compared with the model control group (*p* = 0.0217), but without significant difference compared with the normal control group (*p* = 0.6851). The number of fecal Bifidobacterium and *Lactobacillus* in the model group were significantly decreased compared with the normal control group (*p* < 0.0001); While they were significantly increased in the MMF group compared with the model control group and normal control group (*p* = 0.009; *p* < 0.0001). The amount of fecal pathogenic flagellated bacteria in the model group was significantly increased compared with the normal control group (*p* = 0.001); However it was significantly reduced in MMF group compared with the model group (*p* = 0.026), which has no statistically difference with the normal control group (*p* = 0.6486). The content of flagellin in colonic mucosa was significantly increased in the model group when compared with the normal control group (*p* < 0.0001), and it was decreased in MMF group compared with the normal control group (*p* < 0.0001), but there was no statistical difference with the normal control group (*p* = 0.6545). The expression level of TLR5 protein in colonic mucosa of rat was significantly increased in model control group compared with the normal control group (*p* = 0.0034), However, it was significantly decreased in MMF group compared with normal control group (*p* = 0.0019), but it was no statistical difference with the normal control group (*p* = 0.7519).

**Conclusion:** MMF can reduce visceral hypersensitivity by decreasing the content of pathogenic flagellated bacteria and their flagellin and inhibiting its specific receptor TLR5 protein expression in colonic mucosa in IBS-D rats.

## 1 Introduction

Irritable bowel syndrome (IBS) is a functional bowel disorder characterized by abdominal pain or discomfort with a change in bowel habits without organic disease. The most common one is the diarrhea dominant Irritable bowel syndrome IBS-D (Oka et al., 2020). The pathogenesis of IBS-D is complex and its core pathological change is knowen as “visceral hypersensitivity” nowadays. However, the mechanism of this pathological process has not yet been clarified. In recent years, many studies have shown that imbalance of intestinal flora and increased colonization of harmful bacteria are one of the important pathogenesis and clinical manifestations of IBS-D patients, which are considered to be the etiological factors of visceral sensitivity in IBS-D patients (De Palma et al., 2017).

Massa Medicata Fermentata (MMF), a traditional Chinese medicine, was found to have a effect on relieving visceral hypersensitivity symptoms such as abdominal tension, abdominal pain, and diarrhea in IBS-D patients during our clinical medication. Modern pharmacological studies have shown that MMF has anti-inflammatory and intestinal mucosal barrier repair effects (Bose et al., 2012). Some studies also have shown that MMF has the effects of regulating intestinal bacteria polymorphism, reducing harmful bacteria colonization, and increasing the number of probiotic bacteria in weaning piglets (Wang et al., 2018). However, the specific regulation of MMF on intestinal bacteria and the mechanism of its alleviation effect on visceral hypersensitivity in IBS-D have not been elucidated in detail. Therefore, this study aims to observe the mechanism of the influence of MMF on visceral hypersensitivity in IBS-D.

## 2 Materials and methods

This project was approved by the Ethics Committee of Wenzhou Hospital of Integrated Traditional Chinese and Western Medicine (2022-K001).

### 2.1 Experimental animals

Thirty SD male rats, weighing 250 ± 10 g (provided by the Laboratory Animal Center of Zhejiang Chinese Medical University), without deformity, trauma, and skin infection. They were fed with normal diet *ad libitum*, given 12 h light per day, and subjected to experimental procedures after 1 week of adaptive feeding.

### 2.2 Instruments and reagents

#### 2.2 1 Main instruments

The main instruments used in this study are the following: 8F urinary catheter (diameter 2 mm, maximum balloon capacity 3 ml, maximum diameter 2 cm) was used as colorectal balloon dilatation catheter (produced by Zhejiang Hengkang Medical Instrument Co. Ltd.); AMPure XP Beads (BECKMAN), Microplate reader (BioTek), MiSeq sequencer (Illumina), microplate reader (Bio-Rad) were each used in relevant experimental operations.

#### 2.2 2 Main reagents

The main reagent materials used in this study are the following: glacial acetic acid (Sinopharm 10000208), Q5 ^®^ High-Fidelity DNA Polymerase (NEB), Quant-iT PicoGreen dsDNA Assay Kit (Invitrogen), TruSeq Nano DNA LT Library Prep Kit, MiSeq Reagent Kit V3 (Illumina), High Sensitivity DNA Kit (Agilent), VAHTS DNA Clean Beads (Vazyme), Rat Flagellin Antibody (FP-Ab) ELISA Assay Kit (Jianglai), RIPA Lysate (Biyuntian), BCA Protein Concentration Assay Kit (Biofroxx) were used in the relevant experimental projects in strict accordance with the instructions.

In this project, the Illumina MiSeq/NovaSeq platform was used for double-end (Paired-end) sequencing of community DNA fragments. The classification-sklearn algorithm in QIIME2 (2019.4) was used for the bacterial 16S rRNA gene counting. Qiime feature list was used to refine the function, and then the ASV/OTU table was extracted, and the extraction depth was set to 95% of the minimum sample sequence volume. Based on the distribution of ASV/OTU in different samples, the Alpha diversity level of each sample was assessed, and at the ASV/OTU level, the distance matrix of each sample was calculated, and the beta diversity differences and the significance of differences between different samples (groups) were measured by various unsupervised sorting and clustering means, combined with the corresponding statistical tests. PERMANOVA, Anosim and Permdisp algorithms were called to test the significance of differences between the subgroups.

### 2.3 Experimental methods

#### 2.3.1 Grouping

The rats were randomly divided into normal control group (*n* = 10), MMF group (*n* = 10) and model control group (*n* = 10) by random number table. Environment setting: room temperature 22–24°C, humidity <60%, noise <50db, and fed with normal drinking water.

#### 2.3.2 Model establishment

Acetic acid enema combined with restraint stress was used to establish IBS-D visceral hypersensitivity model in the model control group and MMF group (La et al., 2003; Bourdu et al., 2005; Zhuang et al., 2015); Corresponding saline enema treatment was performed in the normal control group.

The specific methods were as follows: Before the experiment, the rats in the MMF group were fasted for 24 h before the experiment. After anesthesia with Barbital sodium (40 mg/kg), a silicone tube with a tail end connected to a syringe was inserted 8 cm from the anus, and 1 ml of acetic acid at a concentration of 4% was injected into the colon. After removing the silicone tube, the anus was compressed by hand and the tail of the rats was elevated for 30 s. Then the colon was washed once with 1 ml of 0.01 mol/L PBS solution. The rats were returned to their cages for free movement after the manipulation.

Restraint stress was performed from the 7th to 9th day of the experiment: the rats were placed in a specific restraint device (a self-made Nongfu spring mineral water bottle with the mouth removed, and the length of the bottle was subject to the range of the front and rear limbs of the rats), and their limb activities were restricted without affecting their breathing. Rats were returned to their cages after being restrained for 3 h. This process was repeated for 3 days. Normal control group were given no restraint operation.

#### 2.3.3 Pharmacological intervention

On the 10th day of the experiment, the rats in the drug intervention group were given MMF granules (chengdu - China resources sanjiu) as MMF aqueous solvent with crude drug content of 0.4 g/ml, and given intragastric administration of 2 g/kg for 7 days. Model control group and normal control group were given 0.9% normal saline equal volume gavage for 7 days.

#### 2.3.4 Model assessment

##### 2.3.4.1 Measurement of body weight of rats

Changes in body weight of rats in each group were observed to assess nutritional status.

##### 2.3.4.2 Measurement of intestinal motility in rats

On the 17th day of the experiment, after the rats were adapted to the fixator (self-made restraint device described above) for 30 min, the number of defecation and fecal characteristics (waterly, soft, and hard) within 1 h from 9:00 to 10:00 were counted in each group to evaluate the intestinal motility changes in each group.

##### 2.3.4.3 Visceral sensitivity measurements in rats

On the 17th day of the experiment, the abdominal withdrawal reflex (AWR) test was performed to assess the viscreral sensitivity of the rats: the rats were placed in a fixator (the self-made restraint device described above), and an 8F urinary catheter was inserted into the anus of the rats to make the catheter balloon site about 2 cm away from the anal hilum, and fixed with an application using the figure-of-8 fixation method. After the rats were adapted for 30 min, saline was injected into the catheter balloon, and according to the internationally abdominal withdrawal reflex (AWR) test scoring criteria (Williams et al., 1988; Chaer et al., 2000), recorded the volume (ml) of saline injected into the balloon when the score was four points (acute contraction of abdominal muscles, arched abdomen, and lifting of abdomen and perineum off the ground). Each rat was detected 3 times and the average value was taken.

#### 2.3.5.16 s RNA sequencing of rat feces

On the 17th day of the experiment, feces samples were extracted from the rats for second-generation sequencing, and species composition analysis, species difference and marker species analysis, Alpha diversity analysis, Beta diversity analysis were performed.

#### 2.3.6 Sample collection

On the 18th day of the experiment, the rats received 3% pentobarbital (40 mg/kg) anesthesia by intraperitoneal injection, and 2 cm of full-thickness colonic tissue 6 cm from the anus was taken from the abdominal cavity of rats by dissection, all tissues were cut longitudinally, washed with normal saline, then divided into two parts, and soaked in 10% neutral formalin or liquid nitrogen, respectively. Finally, all rats were sacrificed by cervical dislocation.

#### 2.3.7 Sample processing: TLR5 protein extraction and detection in colonic mucosa by western blot

According to the amount used, add 10 ul of PMSF to each 1 ml of RIPA, so that the final concentration of PMSF is 1 mm, and mix well for later use. After tissue mincing, the lysate was added at a ratio of 150–250 ul of lysate per 20 mg of tissue. For cell samples, remove the culture medium and wash once with PBS, normal saline, or serum-free culture medium. Lysates were added at a ratio of 150–250 ul of lysate per well of cell amount in a 6-well plate. The lysate was brought into full contact with the cells by pipetting several times. The lysed samples were centrifuged at 10,000–14,000 g for 3–5 min, and the supernatant was taken for subsequent protein concentration determination. Sample protein concentrations were determined using a BCA protein assay kit. A loading buffer was added to each sample to adjust the protein concentration to 500 μg/ml. The sample size was determined according to the sample concentration to ensure that the total protein load of each sample was about 40 μg, which was consistent as far as possible. An appropriate amount of × 2 protein loading buffer was added to the protein sample and a boiling water bath was performed at 95–100°C for 5 min. The transferred membrane was blocked with blocking solution at room temperature for 1 h. The blocking solution was removed, and the primary antibody diluted with primary antibody diluent was added overnight at 4°C. The diluted primary antibody was recovered and washed three times for 5 min each with TBST. Add the secondary antibody diluted with the secondary antibody diluent (dilution ratio 1:10000), incubate at room temperature for 30 min, wash four times with TBST on a shaker at room temperature for 5 min each time. A freshly prepared mixture of ECL (A: B = 1:1) was added dropwise to the protein side of the membrane and exposed in a dark room. Adjust the exposure conditions according to different light intensities for development and fixation.

#### 2.3.8 Sample processing: Colonic mucosal flagellin content detection by ELISA

Set 10 wells of standard wells on the enzyme-labeled coated plate, add 100 μl of standard to the first and second wells, then add 50 μl of standard diluent to the first and second wells, and mix well; then take 100 μl from the first and second wells to the third and fourth wells, respectively, and then add 50 μl of standard diluent to the third and fourth wells, and mix well; then discard 50 μl of standard diluent from the third and fourth wells, then take 50 μl of standard diluent to the fifth and sixth wells, respectively, and mix well; after mixing, take 50 μl of standard diluent from the fifth and sixth wells to the seventh and eighth wells, respectively, and then add 50 μl of standard diluent to the seventh and eighth wells, respectively, and then add 50 μl of standard diluent to the seventh and eighth wells, and mix well; then take 50 μl of standard diluent from the seventh and eighth wells to the ninth and eighth wells, respectively, and then add 50 μl of standard diluent to the ninth and eighth wells, and mix well. After dilution, the injection volume of each well is 50 μl, and the concentrations are 36, 24, 12, 6, and 3 ng/ml, respectively). Respectively set blank wells (blank control wells are not added with sample and microplate reagent, and the other steps are the same) and sample wells to be tested. Add 40 μl of sample diluent to the sample well to be tested on the enzyme-labeled coated plate, and then add 10 μl of sample to be tested (the final dilution of sample is 5 times). Add sample to the bottom of the well of the microtiter plate, try not to touch the well wall, and gently shake to mix well. Incubate the plate at 37°C for 30 min after sealing the plate with a microplate sealer. Use distilled water to dilute 30 (20X of 48T) times concentrated washing solution for future use. Carefully remove the microplate sealer, discard the liquid, shake to dryness, fill each well with washing solution, allow one to stand for 30 s and then discard, repeat this procedure for 5 times, and pat to dryness. Add 50 μl of enzyme-labeled reagent to each well, except for blank wells. After incubation and washing, first add 50 μl of chromogenic agent A to each well, then add 50 μl of chromogenic agent B, gently shake and mix well, develop the color at 37°C for 15 min in the dark, add 50 μl of stop solution to each well, terminate the reaction, measure the absorbance (OD value) of each well in sequence with blank air conditioner zero and 450 nm wavelength. The assay should be performed within 15 min after addition of the stop solution.

#### 2.3.9 Statistical methods

Measurement data were expressed as mean ± standard deviation (‾χ ± S), and counting data were expressed as frequency (n) and percentage (%). After the test of row independence, normality and homogeneity of variance, single-factor multiple analysis of variance (ANOVA) or multiple rank sum test (Kruskal–Wallis) were used for intra-group comparison according to the specific situation. When there were intra-group differences, Bonferroni method was used for inter-group comparison. SPSS26.0 statistical software was used for data statistical analysis. *p* < 0.05 was considered statistically significant.

## 3 Results

### 3.1 General conditions of rats

After acetic acid enema modeling, rats in the MMF group and model control group showed restlessness, loose watery stools, perianal contamination, residual feces, increased water consuming, and significantly reduced food intake on days 1–2. From the 4th to 5th day, the water in fecal decreased in those two groups, presenting mainly soft stools. On the 7th day, when restraint stress started, they almost all had soft stools, with occasional granular feces.

After the completion of acetic acid enema combined with the restraint stress operation, the rats showed apathy, hair shrugging, decreased activity, unresponsiveness, watery stool, soft stool, perianal residual stool, significantly reduced eating and drinking water; During MMF gavage, the rats in the MMF group showed decreased self-drinking behavior, increased rodent action, and their response became sluggish.

Rats in the normal control group did not see the above abnormal behaviors.

### 3.2 Body weight changes in rats

The weight of rats before and after the experiment was observed to evaluate the nutritional status of rats statistically.

At the end of the experiment, the body weight of rats in the model control group was significantly lower than that in the normal control group and MMF group (*p* = 0.0235; *p* = 0.0429). There was no significant difference between the MMF group and the normal control group (*p* = 0.8783). It suggests that the nutritional status of rats in the model control group decreased, however, it was not adversely affected in the MMF group. See Table 1 for details.

**TABLE 1:** Weights of rats.

	Before modeling/g	After modeling/g
NC group	176.75 ± 11.59	242.75 ± 6.70
MMF group	177.75 ± 4.11	240.25 ± 6.65
Model group	175.5 ± 8.06	227.75 ± 3.30*#

*p < 0.05 *vs.* NC. ^#^ *p* < 0.05 *vs.* MMF.

### 3.3 Intestinal motility result in rats

The defecation of rats in each group were observed before and after the experiment to statistically assess the intestinal motility changes.

After modeling, the number of soft and watery stools in the model control group was significantly higher than that in the normal control group as well as the MMF group (*p* = 0.0303; *p* = 0.0303), suggesting that their intestinal motility was significantly higher in the model control group after IBS-D visceral sensitivity modeling.

**FIGURE 1:**
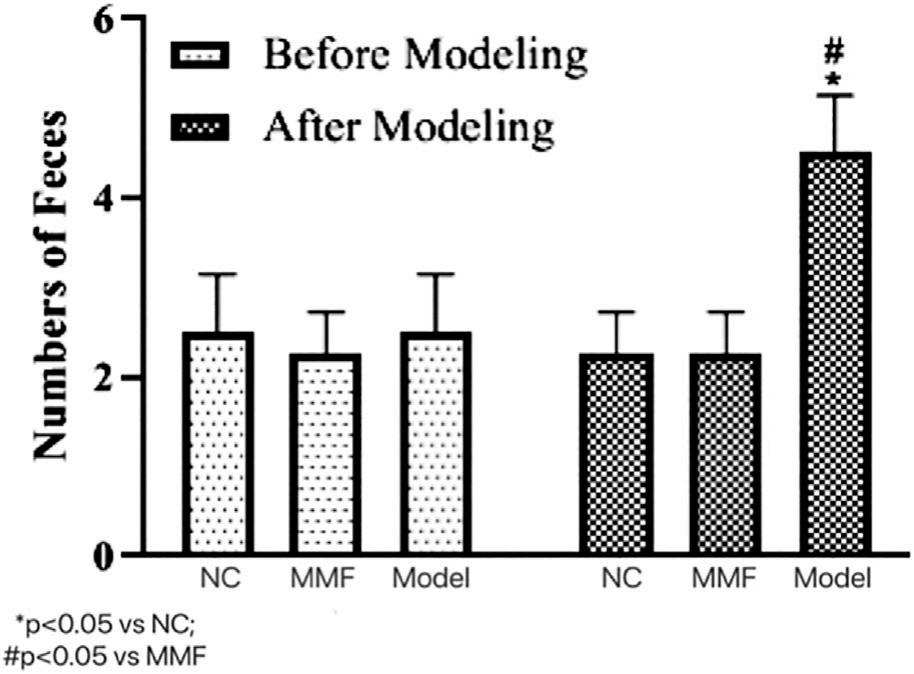
Effects of IBS-D modeling and MMF treatment on fecal pellet output. On the 17th day of the experiment, between 9 and 10 a.m.

And there was no significant difference between the MMF group and the normal control group (*p* > 0.9999), suggesting that MMF can relieve the symptoms of intestinal hyperkinetic in IBS-D rats. See Figure 1 for details.

### 3.4 The visceral sensitivity result in rats

The colorectal balloon inflation volume were compared to observe the changes of intestinal sensitivity of rats when bdominal withdrawal reflex (AWR) test score = 4 in each group.

After modeling, the pressure tolerance of balloon dilation in colon of rats in model control group was significantly decreased when compared with the normal control group (*p* = 0.0061), suggesting that the visceral sensitivity of rats in the model control group was increased after IBS-D visceral sensitivity modeling.

**FIGURE 2:**
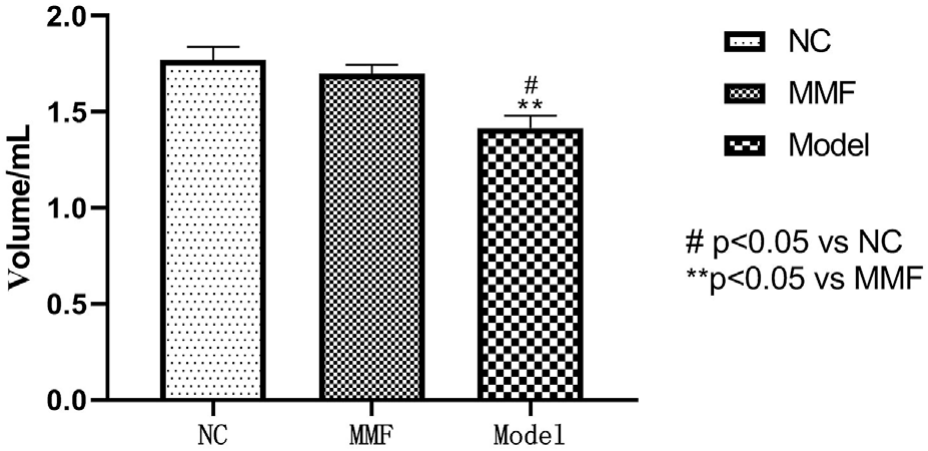
Differences in colorectal balloon inflation volume (ml) among the three groups at AWR = 4

There was a statistical difference of colonic balloon dilation volume between the MMF group and the model control group (*p* = 0.0217), but there was no significant statistical difference between the MMF group and the normal control group (*p* = 0.6851). Suggesting that MMF drug intervention can relieve visceral hypersensitivity in IBS-D rats. See Figure 2 for details.

### 3.5 Rresults of 16S rRNA sequencing of feces in rats

**FIGURE 3:**
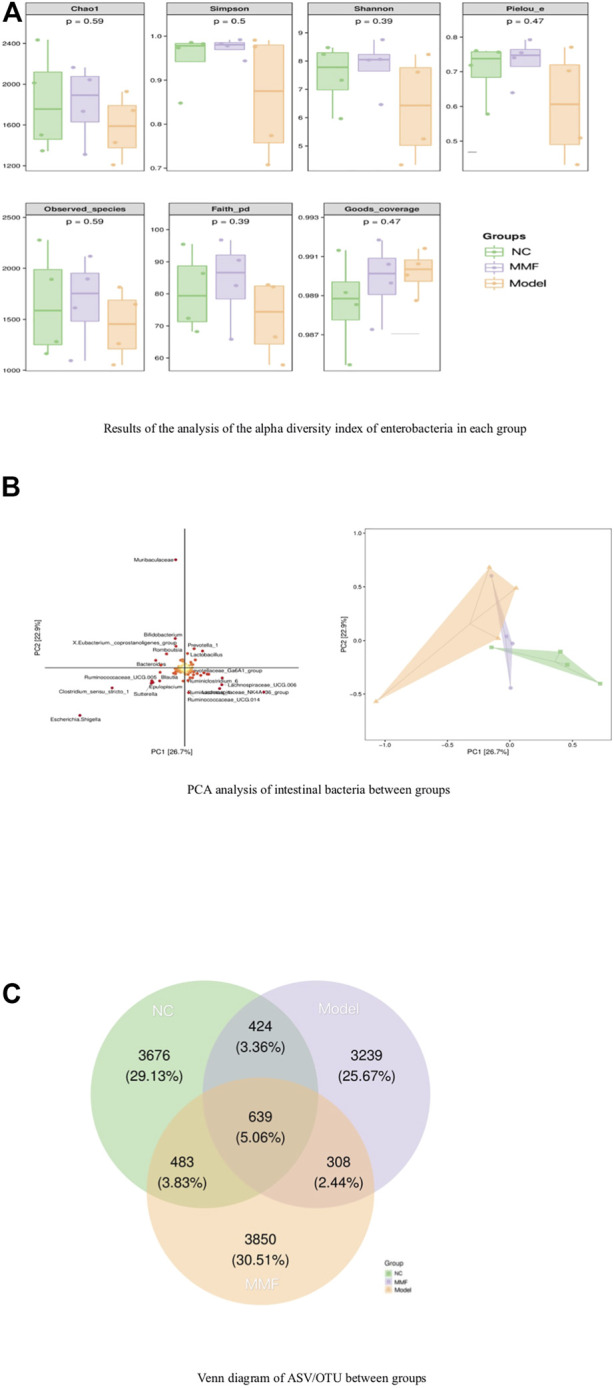
Analysis of species differences among groups. **(A)** Results of the analysis of the alpha diversity index of enterobacteria in each group. **(B)** PCA analysis of intestinal bacteria between groups. **(C)** Venn diagram of ASV/OTU between groups.

The results of 16S rRNA sequencing of feces in rats showed that the number of microbial communities was similar at each taxonomic level between the normal control group and the MMF group byαdiversity analysis, and the number of microbial communities in the model control group was less than that in the normal control group and the MMF group, especially at the level of families and genera level (*p* = 0.0317 *p* = 0.0411; *p* = 0.0315 *p* = 0.0397). It is suggested that the diversity of intestinal bacteria in rats decreased after IBS-D modeling, and the abundance of intestinal bacteria in rats recovered after drug intervention. As detailed in Figure 3A.

Analysis byβdiversity indicated significant species differences between model control rats and normal control and drug intervention groups, as detailed in Figure 3B.

To further explore which species’ differential distributions are responsible for these differences, we used the ASV/OTU abundance tables to produce Venn diagrams for the analysis. From Figure fig3.C, the number and percentage of assembled and unique flora among the groups can be seen. The control and model groups shared 1063 colonies (8.42%), the MMF and model groups shared 947 colonies (7.5%), and the control and MMF groups shared 1122 colonies (8.89%). The control and MMF groups shared the most flora. Among them, the MMF group had 3850 unique flora, accounting for 30.51%, which was the most among the three groups.

### 3.6 Results of intestinal beneficial bacteria in rats

The number of Bifidobacterium and *Lactobacillus* in feces of rats in model control group decreased significantly compared with normal control group (*p* = 0.0012; *p* = 0.0002). While they were found increased in the MMF group when compared with the model control group and normal control group (*p* = 0.009; *p* < 0.0001); (*p* < 0.0001; *p* < 0.0001).

**FIGURE 4:**
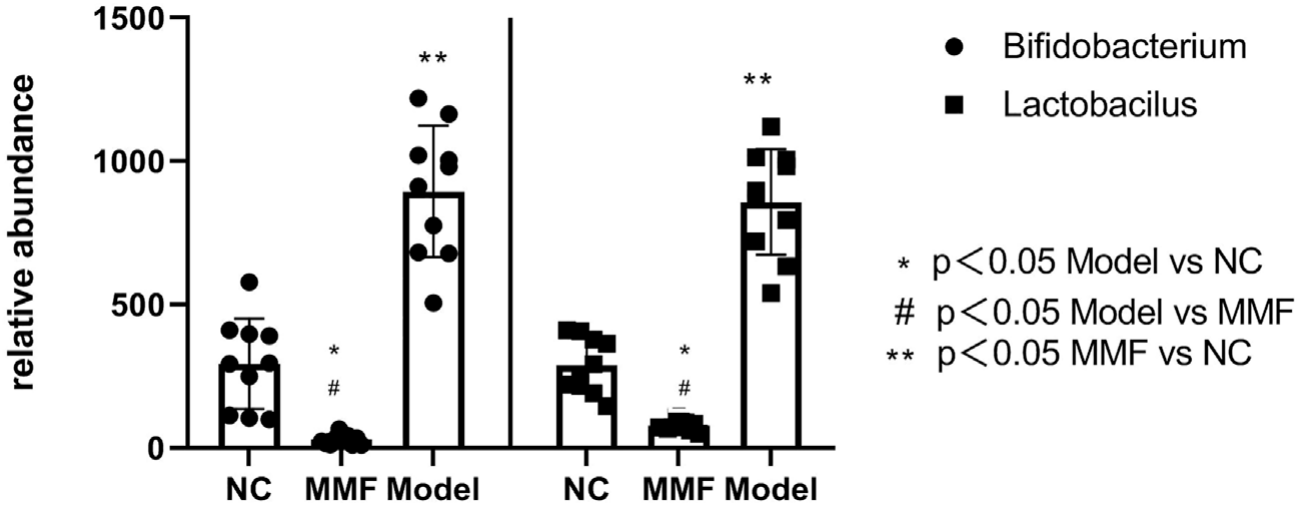
Contents of Bifidobacterium and *Lactobacillus* in feces of rats in each group.

It suggests that the contents of intestinal probiotics decreased after IBS-D visceral sensitivity modeling. However, this situation can be ameliorated by MMF intervention. See Figure 4 for details.

### 3.7 Result of intestinal pathogenic flagella bacteria in rats

Compared with the normal control group, the content of pathogenic flagella bacteria such as *Escherichia*, *Klebsiella* and Pseudomonadales in the Enterobacteriaceae family of feces was significantly increased in the model control group (*p* = 0.001); However it was significantly reduced in the MMF group compared with the model group (*p* = 0.026); which was not statistically different compared with the normal control group (*p* = 0.6486).

**FIGURE 5:**
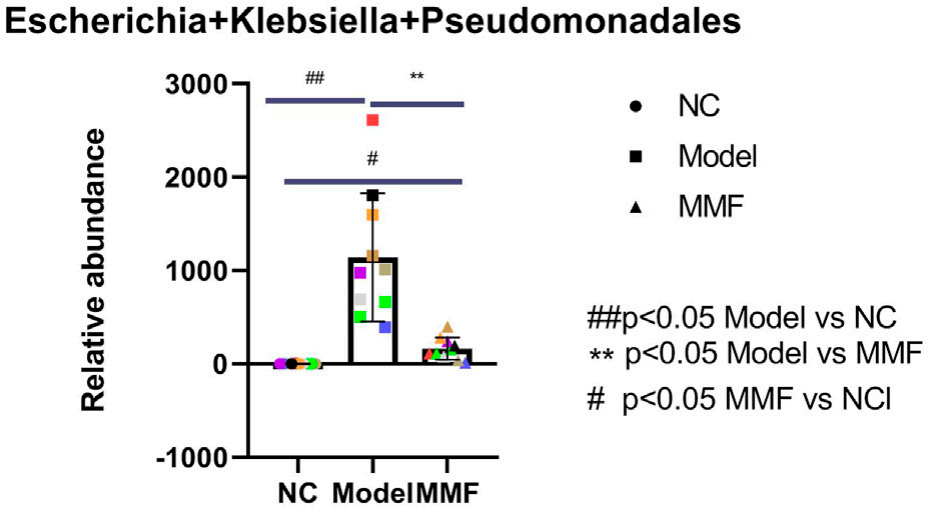
Content of pathogenic flagellated bacterias in feces of rats in each group.

It suggests that the pathogenic flagella bacteria in the intestine of rats increased after IBS-D visceral sensitivity modeling, while the intervention of MMF can ameliorate this situation. See Figure 5 for detail.

### 3.8 Result of flagellin in colonic mucosa of rat

The content of flagellin in colonic mucosa was significantly increased in the model group when compared with the normal control group (*p* < 0.0001), while it was decreased in the MMF group when compared with the model control group (*p* < 0.0001), but it was no statistical difference compared with the normal control group (*p* = 0.6545).

**FIGURE 6:**
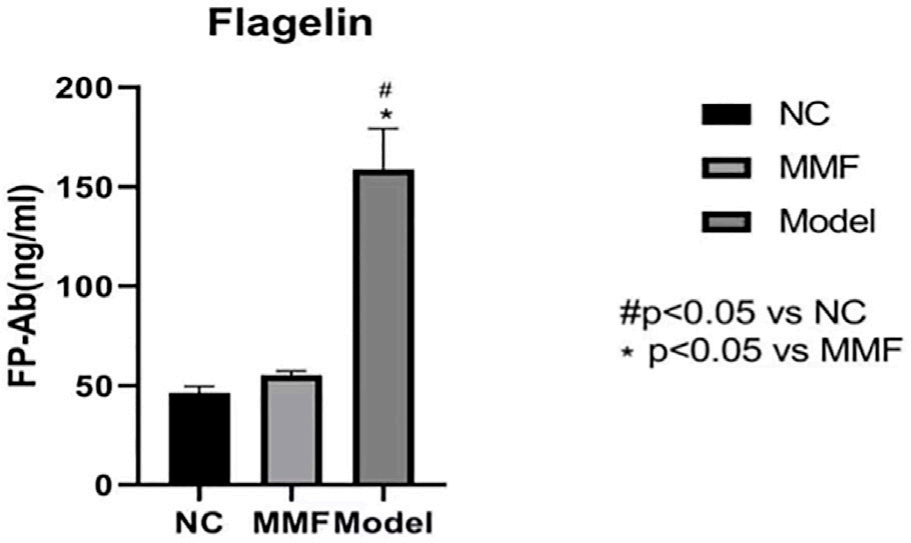
Flagellin content in colonic mucosa of rats in each group.

These results suggest that the flagellin in colonic mucosa of rats increased after IBS-D visceral sensitivity modeling, while it could be reduced by MMF intervention, as shown in Figure 6.

### 3.9 Result of TLR5 protein expression in colonic mucosa of rat

Compared with the normal control group, the expression level of TLR5 protein in colonic mucosa of rats were significantly increased in model control group (*p* = 0.0034), However, it was significantly decreased in the MMF group compared with normal control group (*p* = 0.0019), but it was no statistical difference when compared with the normal control group (*p* = 0.7519).

**FIGURE 7:**
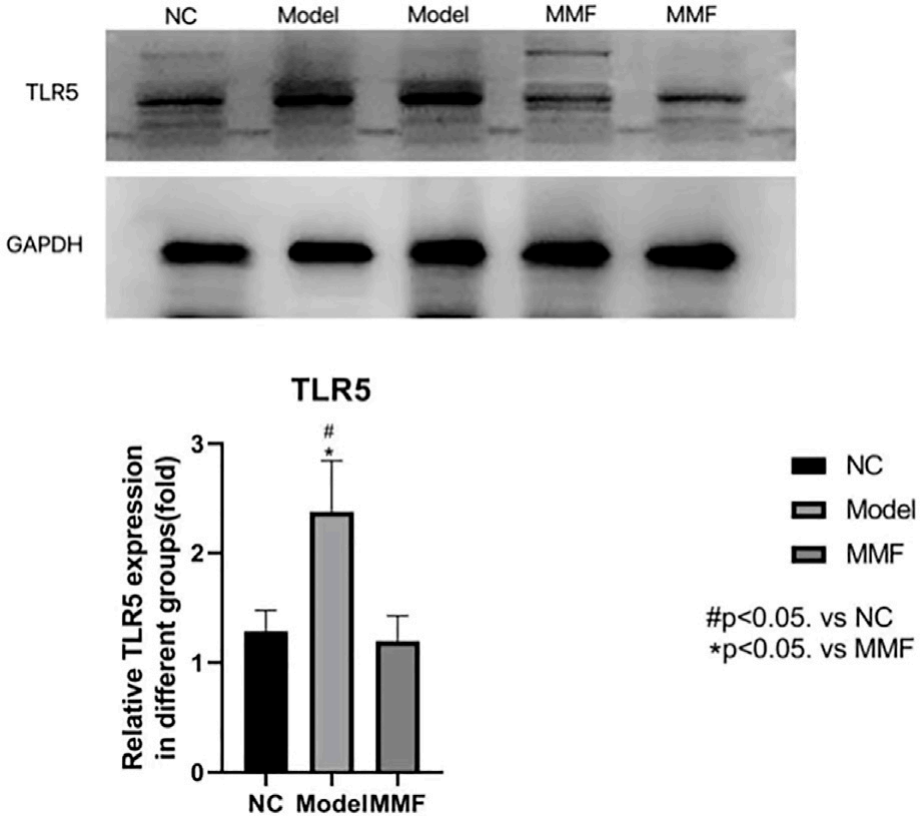
Result of TLR5 Protein Expression in colonic mucosa of rats in each group.

The result suggests that TLR5 protein expression increased in the colonic mucosa of rats after IBS-D visceral sensitivity modeling and can be decreased after intervention with MMF, as detailed in Figure 7.

## 4 Discussion

The pathogenesis of IBS-D is complex and not yet fully clarified. Some pathologic changes such as the intestinal flora imbalance, the chronic inflammatory response of the colonic mucosa, the abnormal intestinal mucosal immune response to colonic mucosa and the increased visceral sensitivity in the intestine are common in patients with IBS-D (Kyloh et al., 2011). In recent years, many studies have shown that the imbalance of intestinal flora is one of the important pathogenesis and clinical manifestations of IBS-D, which is considered to be the etiological factor of IBS-D patients and can aggravated visceral sensitivity symptom of them (De Palma et al., 2017).

Compared with healthy person, IBS-D patients have significant species differences in intestinal flora, accompanied by impaired intestinal mucosal biological barrier, which further induces harmful bacteria colonization. It shows that in IBS-D patients the content of dominant flora was significant decreased and the proportion of harmful flora was increased. The number of *Escherichia coli*, Bacteroidetes and *Clostridium* increased significantly, while the number of bifidobacterium decreased (Pittayanon et al., 2019; Zhong et al., 2019). Jizhong et al. found that there was an excess of *Escherichia coli* in the intestine of IBS-D patients, which could release the corresponding cytokines by affecting the Toll-like receptors of immune cells in the body, thus leading to visceral hypersensitivity in IBS-D patients (Salem et al., 2018). Guo et al. (2016) found that the number of both Portunus and *Bacteroides* in the intestinal mucosa of IBS-D patients was significantly reduced compared with healthy controls, and was positively correlated with diarrhea symptoms (Ji Zhong et al., 2016). Tap et al. (2017) found that the severity of intestinal symptoms in patients with IBS- D was negatively correlated with *Clostridium* or Prevotella, and could not be improved by dietary modification or the use of other drugs (Guo et al., 2016). Those studies suggest that intestinal bacterial changes play an important role in the pathogenesis of IBS-D.

In the IBS-D visceral hypersensitivity model of rats, we found that the diversity of intestinal flora decreased and the species composition changed. The contents of Bifidobacterium and *Lactobacillus* decreased significantly, and the content of *Escherichia coli* increased, those results was consistent with the conclusions of previous studies. However, the mechanism of the effect of altered intestinal flora on visceral sensitivity has not been elucidated.

Our experimental result shows that the amount of pathogenic flagellated bacteria such as *Escherichia*, *Klebsiella*, and Pseudomonadales was significantly increased in the intestinal of IBS-D visceral hypersensitive model rats. At the same time, the content of flagellin in colonic mucosa increased, and the expression of TLR5 protein which is known as the specific receptor for flagellin was also up-regulated in colonic mucosa of IBS-D visceral hypersensitive model rats.

Intestinal flagellated bacteria play an important role in the process of microbial colonization of the intestinal mucosa, secondary infection, and regulation of the immune response of the intestinal mucosa because of their unique flagellar structure. Flagellin is the main functional unit of them, and is associated with the incidence of intestinal infection in IBS-D patients.

Toll-like receptors can induce mucosal immune responses by recognizing different microbial components. Flagellin, the flagellar protein of flagellated bacteria, on the other hand, is the only specific ligand for Toll-like receptors 5 (TLR5), triggering a strong proinflammatory response, and becoming the main stimulator of the intestinal antimicrobial immune response (Steiner, 2007; Fournier et al., 2009; Luo et al., 2014; Mohari et al., 2018). Relevant studies have also confirmed that the TLR5 protein expression is up-regulated in the colonic mucosa of IBS-D patients, accompanied by low-grade inflammatory response and abnormal immune response of colonic mucosal, which were considered closely related to visceral hypersensitivity symptoms (Li et al., 2015; Bielaszewska et al., 2018).

Therefore, we believe that in the intestine of IBS-D visceral hypersensitivity model rats, due to the increased number of pathogenic flagellated bacteria, the flagellin content in the colonic mucosa is increased, which initiated the proinflammatory effect by binding to the specific receptor TLR5 of colonic mucosa, resulting in chronic inflammatory and abnormal immune response in the mucosa and inducing the occurrence of visceral hypersensitivity.

Massa Medicata Fermentata (MMF), a traditional Chinese medicine, is a naturally fermented yeast compound preparation with moderate effects of invigorating the spleen and stomach, and regulating the digestive function. At present, it is commonly used in clinical practice to treat diseases mainly including epigastric fullness, poor appetite, and diarrhea. It is currently widely used in allergic diarrhea, functional diarrhea, chronic diarrhea, and diarrhea-predominant irritable bowel syndrome. Modern pharmacological studies have shown that MMF has the effect of increasing the polymorphism of intestinal bacteria, reducing the number of harmful bacteria, restoring the intestinal microecological balance, increasing the number of beneficial commensals such as intestinal bifidobacteria and bacteroids. Because of the significant antibacterial effect, MMF can inhibit the growth of bacteria such as *Escherichia*, *Salmonella*, and *Staphylococcus aureus* in IBS-D patients, moreover (Bashashati et al., 2014; Fu et al., 2020).

Through this study, we found that MMF could enrich the abundance of intestinal bacteria, increase the number of bifidobacteria and *Lactobacillus* in the intestine of IBS-D visceral hypersensitivity model rats. And the mechanism may be related to the effects that MMF reduced the number of pathogenic flagellated bacteria and their flagellin in the colonic mucosa, which will provides conditions for the proliferation of beneficial symbiotic bacteria. Meanwhile MMF can also inhibit the overexpression of TLR5 protein in the colonic mucosa and relieving the symptoms of visceral hypersensitivity symptoms of IBS-D visceral hypersensitivity model rats.

## 5 Conclusion

In summary, we believe that in the IBS-D visceral hypersensitivity rat model, the diversity of intestinal flora is changed, the number of probiotics such as Bifidobacterium and *Lactobacillus* is reduced, while the content of pathogenic flagellated bacteria were increased, the content of flagellin and the expression of TLR5 on colonic mucosa enhanced, resulting in chronic inflammatory and abnormal immune response in the mucosa and inducing the occurrence of visceral hypersensitivity. MMF can reduce visceral hypersensitivity by decreasing the content of pathogenic flagellated bacteria and their flagellin and inhibiting its specific receptor TLR5 protein expression in colonic mucosa in IBS-D rats. In the next step, we will further investigate the association between pathogenic flagellated bacteria and colonic mucosal immune responses in IBS-D visceral hypersensitivity model rats. To further clarify the pathway between the intestinal flora change and visceral sensitivity in rats.

## Data Availability

The original contributions presented in the study are included in the article/Supplementary Material, further inquiries can be directed to the corresponding author.
